# Intravascular Large B-Cell Lymphoma Diagnosed After Recurrent Stroke: Case Report and Literature Review

**DOI:** 10.3390/neurolint17050068

**Published:** 2025-04-27

**Authors:** Naoko Takaku, Koji Hayashi, Mamiko Sato, Rei Asano, Kouji Hayashi, Toyoaki Miura, Norimichi Shirafuji, Tadanori Hamano, Yasutaka Kobayashi

**Affiliations:** 1 Department of Rehabilitation Medicine, Fukui General Hospital, Fukui 910-8561, Japan; tnaoko18@u-fukui.ac.jp (N.T.);; 2 Department of Neurology, University of Fukui Hospital, Fukui 910-1193, Japanhamano@u-fukui.ac.jp (T.H.); 3 Graduate School of Health Science, Fukui Health Science University, Fukui 910-3190, Japan; khayashi@fukui-hsu.ac.jp (K.H.); yasutaka_k@fukui-hsu.ac.jp (Y.K.)

**Keywords:** lymphoma, large B-cell, intravascular, cerebral infarction, stroke

## Abstract

**Background/Objectives**: We describe a case of intravascular large B-cell lymphoma (IVLBCL) presenting with recurrent cerebral infarctions and review similar reported cases. Our aim is to explore potential early diagnostic markers and discuss their prognostic implications. **Methods/Results**: A 79-year-old man with a history of hypertension, hyperuricemia, and postoperative bladder cancer presented with five to six cerebral infarctions over an 11-month period, despite successive changes in antiplatelet and anticoagulant medications. Neurological examination revealed decreased pain sensation, bilateral hearing loss, and right thenar atrophy. Laboratory studies showed elevated inflammatory markers and soluble IL-2 receptor. CSF analysis revealed elevated protein, β2-microglobulin, IL-6, and IL-10 levels. A skin biopsy was performed to investigate suspected IVLBCL. Histopathological examination of the skin biopsy revealed large pleomorphic CD20-positive cells within the vasculature, confirming a diagnosis of IVLBCL. The patient was treated with chemotherapy, including dose-adjusted R-CHOP and high-dose methotrexate, and achieved complete remission. No recurrence of cerebral infarction was observed during a two-year follow-up period. **Conclusions**: This case highlights the importance of considering IVLBCL in patients with recurrent strokes of unknown etiology, especially when laboratory or imaging findings suggest systemic involvement. Early recognition and appropriate tissue diagnosis, such as skin biopsy, are essential for timely treatment and favorable prognosis.

## 1. Introduction

Intravascular large B-cell lymphoma (IVLBCL) is a rare form of extranodal non-Hodgkin’s lymphoma characterized by the proliferation of neoplastic lymphoid cells within blood vessels, leading to vessel occlusion and organ dysfunction, while major tissue infiltration is uncommon [1]. This intravascular proliferation leads to vessel occlusion and subsequent organ dysfunction, while the major infiltration of these neoplastic cells into the surrounding tissue parenchyma is typically uncommon [1,2,3]. Previously, IVLBCL was known as malignant angio-endotheliomatosis or angiotropic lymphoma [2]. The disease affects both sexes equally, with a median diagnosis age of 60 to 70 years [1]. The precise incidence of IVLBCL is unknown, but is estimated to be less than 0.5 cases per million inhabitants, emphasizing its rare nature [1].

IVLBCL is distinguished by the presence of large tumor cells within the lumen of blood vessels, with minimal infiltration into surrounding tissues, preventing the formation of external tumors [1,2]. A wide range of symptoms have been reported, including fever, generalized fatigue, neurological symptoms, and respiratory distress, with central nervous system involvement being particularly common [1,2]. The major central nervous system symptoms related to IVLBCL are cognitive impairment/dementia (60.9%), paralysis (22.2%), and seizures (13.4%) [3]. A variety of patterns of central nervous system (CNS) involvement can be observed on imaging, including nonspecific white matter alterations, dispersed microinfarcts, and extensive enhancement [4]. However, the disease has the potential to involve nearly any organ system, depending on where the vasculopathy occurs [1]. Diagnosing IVLBCL is often difficult due to its nonspecific clinical symptoms. A definitive diagnosis frequently requires histopathologic examinations of deep skin biopsies, which may reveal clusters of large, atypical lymphoid cells confined within blood vessel lumens [1]. Additionally, bone marrow aspiration and positron emission tomography–computed tomography (PET–CT) scanning play key roles in both confirming the diagnosis and determining the extent of disease [1].

CNS involvement in IVLBCL exhibits diverse imaging patterns, such as nonspecific white matter changes, scattered microinfarcts, and widespread enhancement [1]. Standard treatment typically includes anthracycline-based chemotherapy combined with rituximab (R-CHOP), which has significantly enhanced response rates and survival outcomes [1,3]. However, due to the frequent CNS involvement and the limited ability of R-CHOP to cross the blood–brain barrier, CNS prophylaxis is often necessary [1]. Although rituximab has improved patient outcomes, IVLBCL remains an aggressive and rapidly advancing type of lymphoma [1]. Notably, studies suggest that patients presenting with cutaneous symptoms may have a better prognosis, likely attributable to earlier detection [1,3]. In this report, we present a unique case of a patient who experienced five or six recurrent strokes in a short period and was diagnosed with IVLBCL via a random skin biopsy. As far as we know, this is the highest number of recurrent strokes in IVLBCL reported to date. In addition, this case did not exhibit any overt skin lesions, which led us to suspect IVLBCL based solely on the clinical presentations and laboratory findings, resulting in a lengthy diagnosis period of one year from the onset. Additionally, we conduct a review of the literature to further elucidate the clinical characteristics of IVLBCL in patients experiencing recurrent strokes. To our knowledge, there have been no previous review reports specifically addressing IVLBCL complicated by recurrent cerebral infarctions, aside from individual case reports. Consequently, the characteristics—including symptoms, differential diagnosis, and etiology—related to recurrent cerebral infarctions associated with IVLBCL remain unclear. This review aims to clarify the clinical features of these cases, including patient outcomes, risk factors, and other relevant details.

## 2. Case Presentation

**Figure 1:**
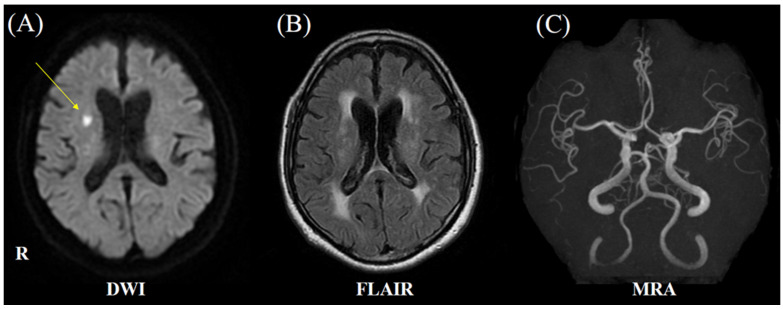
The brain magnetic resonance imaging (MRI) and magnetic resonance angiography (MRA) on first admission (at the initial stroke). (**A**) Diffusion-weighted MRI reveals hyperintensity in the right frontal lobe (yellow arrow). (**B**) T2-weighted fluid-attenuated inversion recovery (FLAIR) MRI demonstrates periventricular leukoaraiosis and deep white matter lesions. (**C**) MRA shows no stenosis or occlusion of major arteries.

A 79-year-old man with a history of hypertension, hyperuricemia, and post-operative bladder cancer (diagnosed at age 67) previously presented to hospital in a wheelchair, reporting a tendency to fall and dysarthria. Around the age of 60, he began experiencing numbness in his hands, which had worsened since turning 70. He was a former smoker, having smoked 40 cigarettes per day from age 20 to 35. Neurological examination revealed dysarthria, bradykinesia, and hyporeflexia in the bilateral Achilles tendon reflexes. Muscle weakness, ataxia, sensory disturbance, and hyperreflexia were not observed. Brain magnetic resonance imaging (MRI) revealed hyperintensity in the right frontal lobe on diffusion-weighted imaging (Figure 1). The patient was diagnosed with a lacunar infarction and was treated with clopidogrel for secondary stroke prevention following acute stroke treatment, which included argatroban. He was discharged home 12 days after his initial visit.

**Figure 2:**
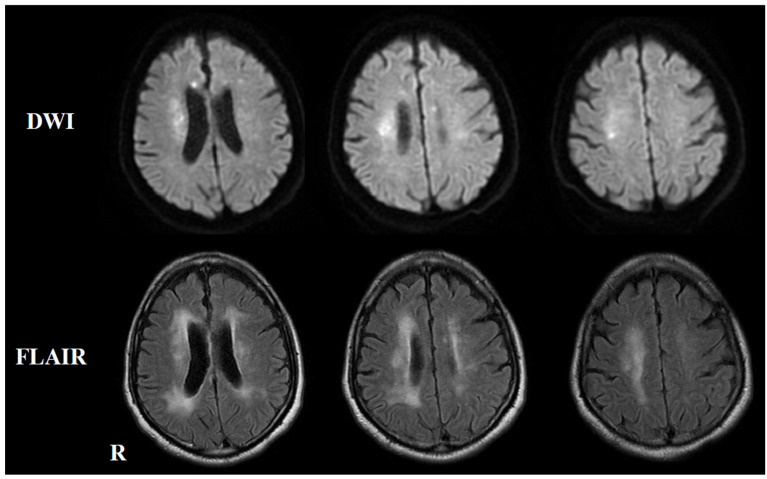
The brain MRI on second admission. The upper panel shows a diffusion-weighted imaging (DWI) brain MRI, while the lower panel displays a T2-FLAIR brain MRI. DWI indicates new hyperintense areas in the frontal lobe and deep white matter near the lateral ventricles. T2-FLAIR shows an expansion of the hyperintense areas in the deep white matter compared to the initial admission.

**Figure 3:**
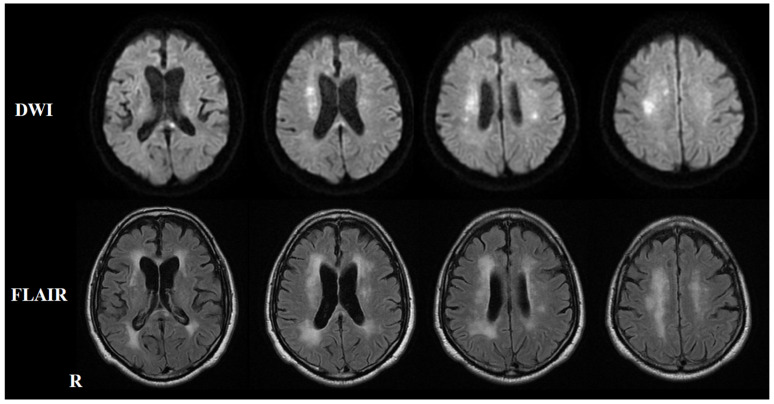
Re-study of brain MRI on second admission after developing cognitive decline. The upper panel shows a diffusion-weighted imaging (DWI) brain MRI, while the lower panel displays a T2-FLAIR brain MRI. DWI reveals hyperintensity in the deep white matter and corpus callosum, with corresponding T2-FLAIR hyperintensity noted in the same regions.

**Figure 4:**
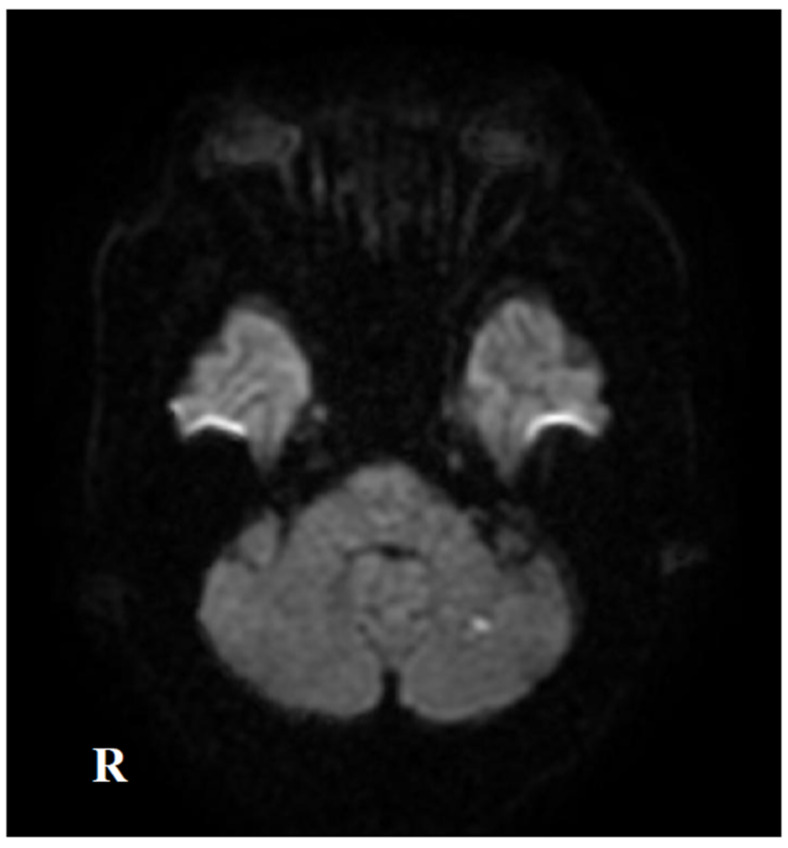
Brain MRI on DWI nine months after the initial onset (fourth admission). Diffusion-weighted brain MRI shows hyperintensity in the left cerebellum.

Two months after the initial symptoms, he was admitted to the previous hospital for dysarthria and dizziness. Brain MRI revealed multiple new infarctions in the frontal lobe and corpus callosum (Figure 2). During hospitalization, he developed cognitive decline. Re-study of the brain MRI revealed multiple new lesions in the deep white matter and corpus callosum (Figure 3). The secondary prevention medication for stroke was switched from clopidogrel to warfarin, and he was discharged from the hospital 29 days after admission. Seven months after the initial onset, he developed hypesthesia in the right foot, which disappeared within one hour. Brain MRI at the previous hospital revealed no new infarctions, and he was diagnosed with a transient ischemic attack (TIA) and hospitalized for 10 days. Nine months after the initial onset, he developed double vision and was admitted to the previous hospital. Neurological examinations revealed medial longitudinal fasciculus syndrome. Brain MRI revealed multiple cerebral infarctions in the pons and cerebellum (Figure 4). Combination therapy of cilostazol and warfarin was selected for the secondary prevention of cerebral infarctions, and he was discharged from the hospital 23 days after admission.

**Figure 5:**
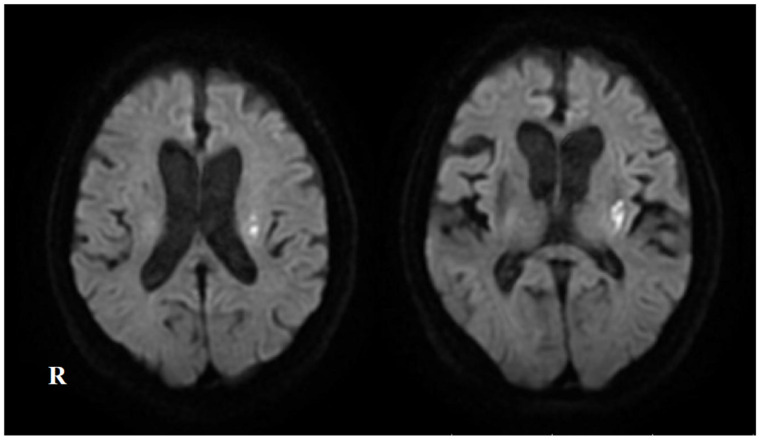
Brain MRI on DWI eleven months after the initial onset (fifth admission). Diffusion-weighted brain MRI shows hyperintensity in the left coronal region.

Eleven months after the initial onset, he developed dysarthria as well as dysesthesia and muscle weakness in the right leg, leading to another admission to the previous hospital. Brain MRI revealed hyperintensity in the right basal ganglia and deep white matter on diffusion-weighted imaging (Figure 5). For the secondary prevention of cerebral infarction, a combination of cilostazol and clopidogrel was prescribed, and he was hospitalized for 26 days.

**Figure 6:**
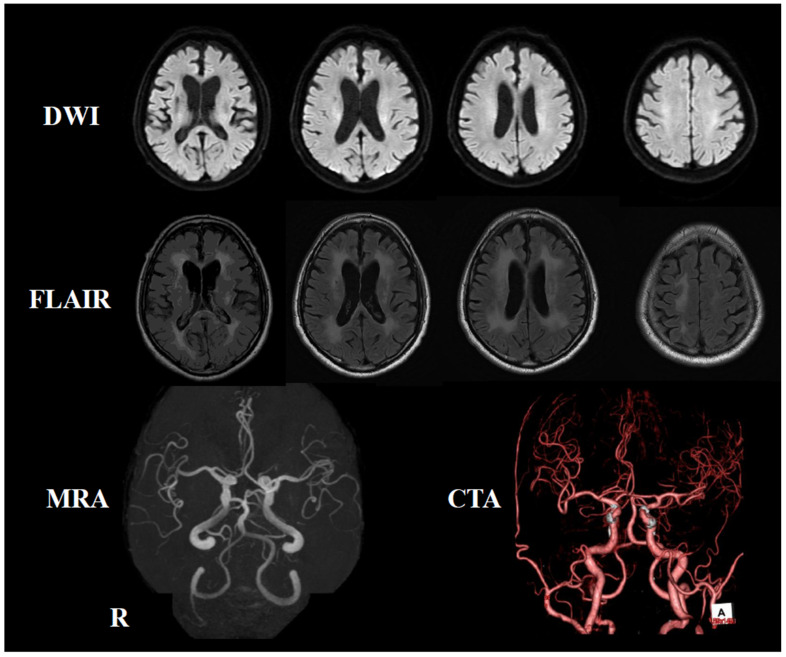
Follow-up MRI study conducted at our hospital. The upper panel shows DWI, the middle panel displays T2-FLAIR, and the lower panel presents MRA and computed tomography angiography (CTA). DWI reveals no hyperintense areas, while T2-FLAIR shows expanding hyperintense areas in the deep white matter. MRA and CTA do not reveal any stenosis in the major cerebral arteries. The “A” in the CTA image stands for “anterior”.

**Figure 7:**
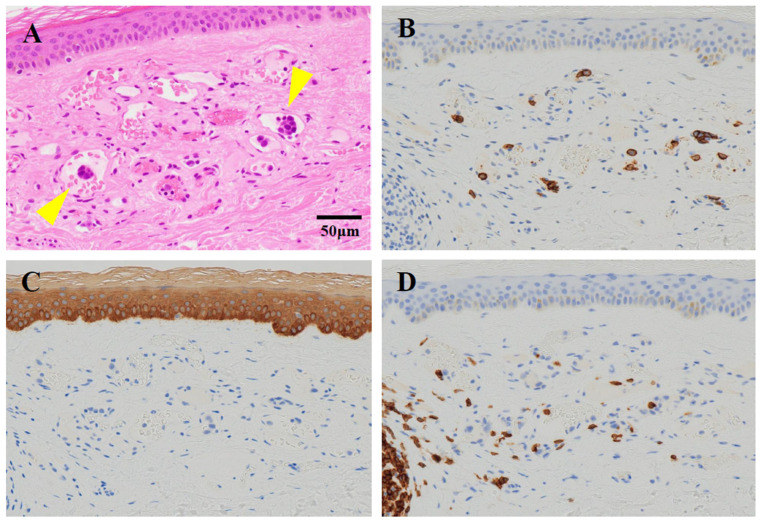
The pathological findings of the skin biopsy. (**A**) On hematoxylin and eosin (H&E) staining, the skin biopsy specimen revealed clusters of large pleomorphic cells with a high nuclear-to-cytoplasmic (N/C) ratio within the superficial dermal blood vessels. (**B**) CD20 immunostaining showed that the cells with a high N/C ratio were positive. (**C**) AE1/AE3 immunostaining indicated that the cells with a high N/C ratio were positive, while the skin epithelial cells were chromatophilic. (**D**) CD3 immunostaining revealed that the cells with a high N/C ratio were positive.

**Figure 8:**
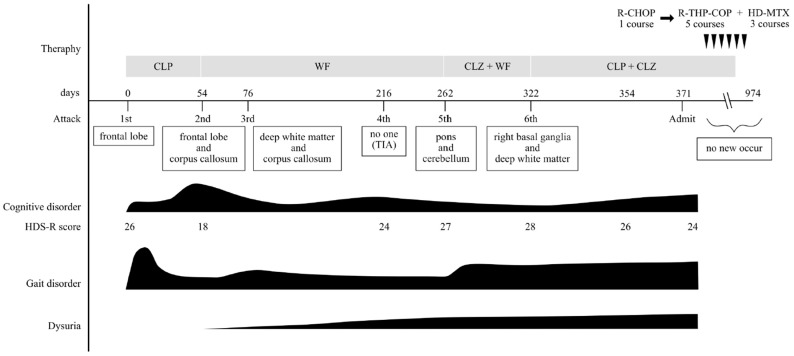
Clinical course of our case. The patient experienced six stroke attacks, including transient ischemic attacks (TIAs). Despite successive changes in antiplatelet and anticoagulant medications, the stroke attacks could not be prevented. Although cognitive function, gait disorders, and dysarthria showed some improvement during the treatment course, they gradually worsened over time. The patient achieved complete remission following chemotherapy, and during the subsequent two years of follow-up, he did not experience any further stroke attacks. Abbreviation: CLP; clopidogrel, WF; warfarin, CLZ; cilostazol, R-THP-COP; rituximab + cyclophosphamide + pirarubicin + vincristine + prednisolone, HDS-R; the Revised Hasegawa’s Dementia Scale.

**Figure 9:**
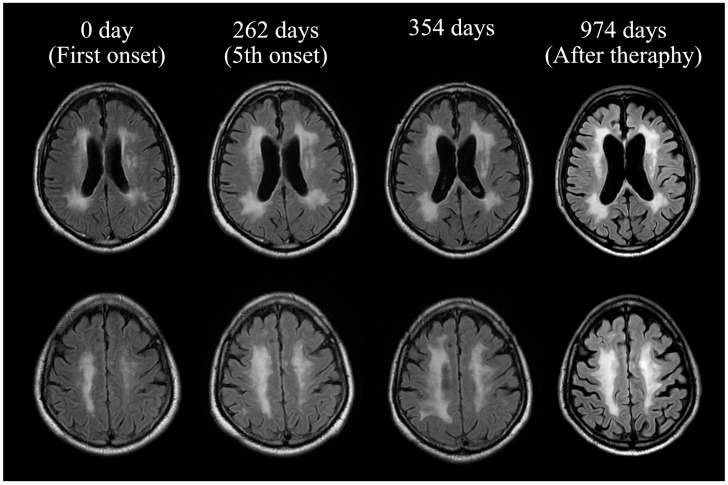
Brain magnetic resonance imaging (MRI) course. T2 fluid-attenuated inversion recovery (FLAIR) MRI revealed expanding hyperintensities in the deep white matter and progressive brain atrophy with each episode of cerebral infarction.

Subsequently, the patient experienced a total of five or six symptomatic attacks, including TIAs, over the course of 11 months. During this time, antiplatelet agents and anticoagulants were switched and used in combination for secondary stroke prevention, but none proved effective. He was admitted to our hospital, requesting a thorough investigation of the causes of his cerebral infarctions. His vital signs on admission were as follows: a temperature of 37.0 °C, blood pressure of 93/59 mmHg, pulse of 67 bpm, and SpO2 of 96%. Physical examination revealed notable dryness and scaling of the skin, but no other significant findings. Neurological examination revealed decreased pain sensation in both frontal lobes, bilateral hearing loss, atrophy of the right thenar eminence, and normal muscle strength except for the bilateral iliopsoas muscles, which were graded four on the Medical Research Council (MRC) scale. Vibratory sensation was absent at the medial malleolus. Increased tendon reflexes were noted in both lower legs. The patient exhibited bilateral clumsiness on the hand pronation test, and their gait was wide-based and unsteady. The Mann test could not be performed due to unsteadiness. Autonomic symptoms were not noted. Blood tests showed elevated levels of erythrocyte sedimentation rate (ESR) at 37 mm/hour, C-reactive protein at 0.76 mg/dL, soluble interleukin-2 receptor (sIL-2R) at 975 IU/mL, ferritin at 363.7 ng/mL, lactate dehydrogenase (LDH) at 398 IU/L, and serum complement at 54.2 units. Conversely, levels of hemoglobin were decreased at 11.6 g/dL, platelet count at 122,000/μL, uric acid at 3.1 mg/dL, cholinesterase at 216 IU/L, and high-density lipoprotein (HDL) cholesterol at 34 mg/dL (Table 1). Autoantibodies, including antinuclear antibodies (ANA), anti-cardiolipin β2 glycoprotein I antibodies (anti-CL β2GPI), lupus anticoagulant, anti-SS-A antibodies, anti-SS-B antibodies, and anti-double-stranded DNA (anti-ds-DNA) antibodies, were not significantly elevated. Cerebrospinal fluid (CSF) analysis revealed a normal cell count but elevated protein levels at 71 mg/dL. In addition, β2-microglobulin was elevated at 2.4 ng/mL (reference range: 0.44–1.24 mg/mL), IL-6 was elevated at 12.6 pg/mL (reference range: <4.3 pg/mL), and IL-10 was elevated at 5.4 pg/mL (reference range: <4.0 pg/mL) in CSF. Holter monitoring revealed no arrhythmias, except for several supraventricular and ventricular premature beats. Carotid artery ultrasound showed mild plaque, but no significant stenosis. Transesophageal echocardiography revealed no evidence of hypertrophy, dilation, or valvular disease. Contrast-enhanced computed tomography (CT) of the thorax and abdomen revealed no malignant findings, lymphadenopathy, or hepatosplenomegaly. The brain MRI revealed scattered old infarcts and magnetic resonance angiography (MRA) showed no significant stenosis in the main arterial branches (Figure 6). Given the elevation of LDH and sIL-2R levels, as well as the increased β2 microglobulin in the CSF, intravascular lymphoma was suspected, prompting the performance of a random skin biopsy. The skin biopsy specimen revealed clusters of large pleomorphic cells with a high nuclear-to-cytoplasmic (N/C) ratio within the superficial dermal blood vessels. Immunostaining showed strong positivity for CD20 and CD3, while AE1/AE3 was negative (Figure 7). These pathological findings indicated that the tumor cells were of B-cell origin rather than epithelial cells. He was ultimately diagnosed with cerebral infarction due to IVLBCL and received chemotherapy in the hematology department. Due to CNS involvement and his advanced age, the initial treatment plan was six courses of R-CHOP with 80% dose adjustment combined with three courses of high-dose methotrexate (HD-MTX). However, severe adverse effects after the first course of R-CHOP led to a change in regimen to R-THP-COP (including rituximab, cyclophosphamide, vincristine, pirarubicin, and prednisolone) from the second course onward. A total of six courses, including the initial R-CHOP, were administered, and HD-MTX was completed as scheduled. He achieved complete remission (CR) and was followed for two years without the recurrence of cerebral infarction. The clinical course of this case in detail is summarized in Figure 8 and Figure 9.

## 3. Discussion

We present a rare case of recurrent cerebral infarction occurring five or six times within 11 months, caused by IVLBCL. Blood tests revealed elevated levels of LDH, sIL-2R, and CRP. CSF analysis showed elevated protein levels, as well as increased levels of IL-6, IL-10, and β2 microglobulin. Brain MRI indicated that the white matter lesions expanded over time. We achieved a diagnosis through a random skin biopsy, which led to effective chemotherapy that prevented further stroke events for at least two years.

IVLBCL is classically classified into Asian and Western types [5]. The Asian type is characterized by common symptoms, including fever with systemic B symptoms [6]. Involvement of the spleen, liver, and bone marrow is typical and is often associated with hemophagocytic syndrome [7]. This variant has the worst prognosis, with a 2-year progression-free survival rate of 56% and a 2-year overall survival rate of 66% following R-CHOP therapy (including rituximab, cyclophosphamide, doxorubicin, vincristine, and prednisone) [5]. On the other hand, the Western type features hallmark symptoms related to the central nervous system, such as rapidly progressive neurologic dysfunction, dementia, recurrent stroke, and peripheral neuropathy [8]. Dermatologic manifestations are more prevalent in the Western variant (17–35%) and include a variety of skin findings, such as erythematous papules, tumors, ulcerated nodules, and tender, indurated, violaceous, or desquamated plaques. The rates of complete remission, overall response, and 3-year overall survival after R-CHOP therapy are 88%, 91%, and 81%, respectively [5]. Based on these findings, IVLBCL with recurrent cerebral infarctions is generally classified as the classical Western type. Additionally, because the Western type preferentially invades the nerves and skin, it is suggested that skin biopsies, including random skin biopsies, may be useful for diagnosing IVLBCL.

Currently, there is no noninvasive method to confirm IVLBCL without a biopsy; however, potential markers that can raise suspicion about the disease are being identified [9]. Potential blood markers for IVLBCL include elevated levels of CRP, ferritin, fibrinogen, LDH, and β2-microglobulin, as well as cytopenia affecting at least one cell line, which may manifest as anemia, lymphopenia, or thrombopenia, which have been noted in most cases. In some instances, liver function tests have revealed cholestasis and elevated transaminases, along with mild hyponatremia and renal failure [9]. CSF abnormalities have also been noted, with increased protein levels observed in 94% of cases and pleocytosis in 71%, although tumor cells have not been detected in cytology. Additionally, some patients may present with elevated levels of CSF LDH, IL-6, IL-10, IL-10/IL-6 ratios, and β2 microglobulin [9,10]. A biopsy is essential for a definitive diagnosis of IVLBCL, as it allows for the identification of tumor cells within the small blood vessels of the tissue [7]. While brain biopsy has the highest diagnostic yield [7], its invasive nature suggests that a random skin biopsy should be considered first for the diagnosis of IVLBCL, unless masses are present in other organs. However, care must be taken with random skin biopsies, as there is a risk of false-negative results. Several factors can contribute to false-negative results in random skin biopsies. First, the prior use of corticosteroids can reduce sensitivity, especially in cases of IVLBCL with central nervous system involvement [11]. Second, low plasma soluble interleukin-2 receptor (sIL-2R) levels, below 3000 U/mL, are associated with a lower tumor positivity rate (45%), requiring multiple biopsies for reliable detection [11]. Finally, biopsy technique plays a role, with punch biopsies demonstrating a lower sensitivity than incisional biopsies due to insufficient sampling depth [12]. To improve the sensitivity of random skin biopsies, obtaining samples from at least three clinically normal-appearing skin sites (e.g., thigh, abdomen, and arm) is recommended [13]. Additionally, including sites with skin lesions in the biopsy can further enhance detection rates, as tumor cells are more abundant in these areas [14]. Therefore, avoiding pre-biopsy corticosteroid administration, which increases the risk of false negatives, is considered beneficial. Likewise, choosing the incisional biopsy method over punch biopsy may contribute to improved detection rates.

FDG-PET/CT is an essential imaging modality for diagnosing IVLBCL [1]. IVLBCL is characterized by a high FDG uptake, which allows PET–CT to identify affected sites with a high sensitivity [1]. PET–CT can help to determine the extent of the disease by identifying affected organ systems and body sites [1]. It can reveal an increased FDG uptake in various locations, including the skin and adrenal glands [15], and can also guide the selection of biopsy sites. Treatment outcomes for IVLBCL have improved since the introduction of rituximab, an anti-CD20 monoclonal antibody, in 2003. A retrospective analysis reported in Japan in 2008 demonstrated that the CR rate with chemotherapy alone for IVLBCL was 51%, whereas the rate increased to 82% with the addition of rituximab [16]. Regarding IVLBCL with CNS involvement, as in the present case, several reports suggest that the suppression of CNS infiltration significantly affects treatment outcomes. In a study evaluating CNS progression or recurrence during treatment in 109 IVLBCL patients who received R-CHOP or CHOP therapy, 17 out of 82 patients without CNS lesions at diagnosis developed CNS involvement during the course of the disease, and 16 of them died [17]. The median overall survival after CNS involvement was 5 months (range: 1–62 months), with a 2-year survival rate of only 12%. In contrast, a comparison between patients treated with R-CHOP alone and those who received CNS-directed therapy including HD-MTX (*n* = 16) revealed a significantly higher 2-year CNS relapse-free survival rate in the CNS-directed therapy group (100% vs. 6.3%, *p* = 0.0191) [18].

Additionally, a phase II trial (the PRIMEUR-IVL study) targeting IVLBCL patients without CNS lesions at diagnosis investigated the efficacy of CNS prophylaxis using HD-MTX and intrathecal chemotherapy. With a median follow-up of 7.1 years (interquartile range: 5.6–8.7), the 5-year progression-free survival was 68% (95% CI: 50–80%) and the 5-year overall survival was 78% (95% CI: 61–89%) among all 37 eligible patients [19].

Based on these findings, CNS involvement was present in the current case and the patient was elderly; therefore, a treatment plan combining six courses of R-CHOP with 80% dose adjustment and three courses of HD-MTX was devised. However, due to severe adverse effects after the first course of R-CHOP, the regimen was changed to R-THP-COP from the second course onward. A total of six courses, including the initial R-CHOP, were administered, and HD-MTX was completed as scheduled. R-THP-COP was shown to be non-inferior to R-CHOP in a retrospective study targeting elderly patients, with a more favorable toxicity profile. The 2-year overall survival was 77.6% in the R-THP-COP group and 79.2% in the R-CHOP group, while 2-year progression-free survival was 68.5% and 78.9%, respectively. No significant differences in response rates were observed between the two groups. Regarding toxicity, the incidence of neutropenia as a manifestation of myelosuppression was 72.4% in the R-THP-COP group and 88.9% in the R-CHOP group [20]. The achievement of CR in our case may be attributed to the combination of R-THP-COP chemotherapy and HD-MTX administration.

Adverse effects of R-CHOP therapy include neutropenia, alopecia, fatigue, taste alteration, nausea, constipation, and insomnia [21]. Additionally, an increased risk of severe neurological symptoms such as facial or ocular muscle paralysis, as well as heart failure, has been reported [22,23]. Adverse effects of HD-MTX therapy include severe myelosuppression, hepatic dysfunction, pulmonary toxicity (such as interstitial pneumonia), renal impairment, and alopecia [24]. Although rare, cirrhosis and pneumonia are serious adverse events requiring caution [24].

To better define the clinical features of IVLBCL cases associated with recurrent cerebral infarctions, we conducted a literature search. We conducted a comprehensive literature search using PubMed and J-STAGE, employing the keywords “Intravascular large B-cell lymphoma, intravascular lymphoma, malignant angio-endotheliomatosis, or angiotropic lymphoma” combined with “Stroke or Cerebral Infarction”. We extracted case reports involving humans, focusing on cases where evidence of multiple cerebral infarctions was presented through head imaging. As a result of our database searches, we identified 20 previously reported cases diagnosed with multiple cerebral infarctions based on brain imaging (Table 2) [25,26,27,28,29,30,31,32,33,34,35,36,37,38,39,40,41,42]. The ages ranged from 42 to 79 years, making our case the oldest. The maximum number of cerebral infarctions described was five. The initial symptoms were primarily typical manifestations of stroke, including paralysis, dysarthria, aphasia, and sensory disturbances; however, cognitive decline, seizures, and fatigue were also noted in a significant number of cases. Symptoms observed after the second occurrence were largely stroke-like, but additional symptoms such as epilepsy, disturbances in consciousness, cognitive dysfunction, and delirium were also documented. LDH was elevated in all reported cases, with levels ranging from 276 to 5843. While some cases presented with negative CRP levels, many demonstrated elevations, with values between 0.08 and 8.34. sIL-2R levels were significantly elevated in all cases, ranging from 975 to 4816. Most cases were diagnosed through skin or brain biopsies. However, in cases where PET scans were conducted, some diagnoses were made through biopsies of other organs, including the kidneys, adrenal glands, and lungs. Furthermore, several cases were not diagnosed during the patients’ lifetimes and were identified posthumously through autopsy [30,32,35,36,40]. This is believed to be due to the challenges of obtaining a diagnosis before death and delays in treatment. The duration from the onset of initial symptoms to pathological diagnosis varied from 1 month to 2 years, although most cases were diagnosed within a few months. Among the 20 cases for which prognosis data were available, 12 resulted in death, while 8 were survivors. We observed that in the group of patients who survived, there was no recurrence of cerebral infarction. Previous studies have reported similar findings, as follows: Hirose et al. found no recurrence after 1 year and 10 months [26], Yamaoka et al. observed no recurrence after 3 months [27], Boslooper et al. reported no recurrence after 7 months [15], and Popiolek et al. found no recurrence after 4 years [41]. Furthermore, upon analyzing the cases that resulted in death, the MRI abnormalities observed were more characteristic of a brain tumor, presenting relatively widespread abnormal signals that did not correspond to the vascular territories typically affected by cerebral infarctions [30,32,34,36,38,39].

The differential diagnosis for IVLBCL presenting with recurrent cerebral infarction is broad, encompassing cerebral infarction of an unknown etiology and other similar conditions. Initial diagnostic efforts should focus on excluding common causes of stroke, such as cardiogenic cerebral embolism, atherothrombotic cerebral infarction, and lacunar infarction. Notably, several previously reported cases, including the current one, were initially misdiagnosed as cardiogenic cerebral embolism, atherothrombotic cerebral infarction, aortagenic embolism, or paradoxical cerebral embolism [25,27,30,35,37]. This review of the literature suggests that IVLBCL frequently occurs in age groups with a high prevalence of atherosclerotic lesions, making it challenging to definitively rule out these common stroke etiologies. Therefore, the recurrence of stroke despite adequate secondary stroke prevention medication should raise the suspicion of IVLBCL. Furthermore, cognitive impairment or seizures are atypical initial presentations of stroke. The presence of these symptoms in a patient experiencing recurrent cerebral infarction may warrant further investigation for IVLBCL.

Vasculitis including infectious and autoimmune disease was listed as a differential diagnosis in some previous reports we reviewed [31,34]. In fact, it has been reported that the head imaging findings of IVLBCL are similar to those of CNS vasculitis [43]. However, it is challenging to exclude the diagnosis of CNS vasculitis, because there are neither specific clinical features nor a classical clinical course, and no blood or imaging investigations can confirm the diagnosis [44]. Additionally, some CNS infections can cause both vasculitis and/or cerebral infarctions, as well as nonspecific imaging findings, including herpes simplex virus, varicella-zoster virus, syphilis, and JC virus [45,46,47,48]. Diagnostic markers such as antibodies, antigens, and genetic tests have been established for some CNS infections, and these should be utilized to rule them out.

Among the previous cases we reviewed, demyelinating disease was a potential differential diagnosis for IVLBCL [29,38]. Demyelinating diseases such as multiple sclerosis and neuromyelitis optica spectrum disorders are relapsing and can lead to recurrent CNS lesions with MRI signal abnormalities. Cerebrospinal fluid studies, including the IgG index and oligoclonal bands, as well as specific antibodies such as anti-aquaporin-4 (anti-AQP4) and anti-myelin oligodendrocyte glycoprotein (anti-MOG) antibodies, may be useful for ruling them out [49,50].

In one previous case, embolism due to infective endocarditis was considered as a unique differential diagnosis for IVLBCL based on the presence of fever, elevated inflammatory markers, and cerebral infarction [15]. Since fever is a common symptom in IVLBCL [1,2] as well as elevated C-reactive protein, an echocardiogram may be necessary to rule out infective endocarditis.

The primary etiology of cerebral infarction in intravascular large B-cell lymphoma (IVLBCL) is attributed to the proliferation of neoplastic lymphoid cells within the lumina of small blood vessels, leading to the occlusion of these vessels and subsequent tissue infarction [15]. This is a defining characteristic of IVLBCL, where lymphoma cells demonstrate a predilection for growth within blood vessel lumens [3]. Specifically, IVLBCL is classified as an extranodal diffuse large B-cell lymphoma characterized by the presence of neoplastic lymphocytes exclusively within the lumina of small vessels, resulting in vessel occlusion and subsequent tissue infarction [15]. These tumor foci can obstruct arterial blood supply to distal locations, causing ischemia in various organs, including the brain [3]. This selective growth within the lumina of small- and medium-sized vessels leads to organ dysfunction due to vascular occlusion [30]. Histological examination in cases of IVLBCL with cerebral involvement has revealed that small vessels (venules, capillaries, and arterioles) are completely or partially occluded by neoplastic cells [42]. This vascular occlusion by tumor cells is understood to cause cerebral infarcts, which are primarily distributed in the territories of small arteries [42]. While damage to vessel walls, potentially leading to degeneration, inflammation, or necrosis of the vascular endothelium, is a possible mechanism related to IVLBCL [28], the primary mechanism for cerebral infarction, as established in the literature, is the physical occlusion of small blood vessels by the accumulation of lymphoma cells within their lumens. This blockage of multiple cerebral blood vessels due to lymphoma proliferation can result in cerebral infarction, possibly leading to recurrent cerebral infarction. Ultimately, these lesions appear on MRI as diffusion-restricted lesions, a pattern consistent with typical cerebral infarction. In summary, in cases of recurrent cerebral infarctions occurring within a short time frame, IVLBCL should be considered in differential diagnoses. To establish a diagnosis, it is essential to thoroughly exclude IVLBCL mimickers such as cerebral infarction, demyelinating diseases, vasculitis, and infections, including infective endocarditis. For the diagnosis of IVLBCL, markers such as LDH, sIL-2R, and inflammatory markers obtained through blood tests can be useful. Additionally, PET scans may be beneficial for detecting lesions in other organs and considering biopsy sites. Brain biopsies and random skin biopsies are essential for establishing a definitive diagnosis. When IVLBCL forms extensive lesions in the brain tissue, it may be strongly associated with mortality. There are cases where neurological symptoms, such as cerebral infarctions, have been resolved with chemotherapy, highlighting the importance of early diagnosis and prompt treatment.

Our case emphasizes that IVLBCL should be considered in patients presenting with recurrent cerebral infarctions occurring within a short timeframe. Notably, increased vigilance is warranted for cases that continue to progress despite modifications in antiplatelet and anticoagulant therapies. In these situations, blood markers such as LDH and sIL-2R, along with CSF markers including IL-6, IL-10, and β2 microglobulin, can strongly indicate the possibility of IVLBCL. Consequently, random skin and brain biopsies may be necessary to confirm the diagnosis and guide appropriate management. Furthermore, the timely initiation of appropriate anti-cancer chemotherapy may help to eliminate the recurrence of cerebral infarction. Thus, early recognition and intervention are crucial for improving patient outcomes in cases of IVLBCL associated with cerebral infarctions.

## 4. Conclusions

Our case report, along with a review of the literature, highlights the importance of considering IVLBCL in the differential diagnosis of patients presenting with recurrent cerebral infarctions, particularly when these events occur within a short timeframe, often within a few months, and persist despite optimized antithrombotic and/or anticoagulant therapy. Elevated blood markers, such as LDH and sIL-2R, along with elevated cerebrospinal fluid markers, including IL-6, IL-10, and β2 microglobulin, should raise suspicion for IVLBCL. Prompt diagnosis through random skin and brain biopsies is crucial for initiating appropriate management, including anti-cancer chemotherapy, which may help to prevent or reduce the recurrence of cerebral infarction and improve patient outcomes. This case, along with the review of the existing literature, emphasizes the need for early recognition and intervention in IVLBCL cases presenting with cerebral infarctions to optimize patient outcomes. For future directions, the development of markers for early diagnosis and optimal chemotherapy regimens to prevent death and neurological sequelae in IVLBCL should be investigated.

## Figures and Tables

**Table 1:** The results of blood tests on admission to our hospital.

Inspection Items	Result	Reference Range
White blood cell count	4100/μL	(3300–8600)
Red blood cell count	347 × 10^4^/μL	(386–492 × 10^4^)
Hemoglobin	11.6 g/dL	(12.1–14.5)
Blood platelet	12.2 × 10^4^/μL	(15.8–34.8)
Total protein	6.8 g/dL	(6.6–8.1)
Albumin	4.1 g/dL	(4.1–5.1)
Glucose	105 mg/dL	(73–109)
Blood urea nitrogen	12 mg/dL	(8.0–20.0)
Creatinine	1.05 mg/dL	(0.46–0.79)
Total bilirubin	0.9 mg/dL	(0.4–1.2)
Aspartate aminotransferase	29 U/L	(13–30)
Alanine aminotransferase	26 U/L	(7–30)
Alkaline phosphatase	72 U/L	(38–113)
Lactate dehydrogenase	398 U/L	(124–222)
γ-glutamyltransferase	48 U/L	(13–64)
Creatine phosphokinase	59 U/L	(41–153)
Choline esterase	216 U/L	(240–486)
Amylase	52 U/L	(44–132)
High-density lipoprotein	34 mg/dL	(40–80)
Sodium	138 mmol/L	(138–145)
Potassium	3.9 mmol/L	(3.6–4.8)
Chlorine	103 mmol/L	(101–108)
C-reactive protein	0.76 mg/dL	(0.00–0.14)
Erythrocyte sedimentation rate	37 mm/h	(<15 mm/h)
Soluble interleukin-2 receptor	975 IU/mL	(157–474)
Ferritin	363.7 ng/mL	(14–304)
Serum complement	54.2 units	(30–45)

**Table 2:** Clinical characteristics of IVLBCL cases diagnosed with recurrent cerebral infarction by brain imaging.

Case	Age/Sex	The Times of Cerebral Infarcts	Initial Symptoms	Initial Diagnosis of the Etiology of Cerebral Infarcts	The Initially Selected Secondary Prevention Medication for Cerebral Infarction	Following Symptoms	LDH (U/L)	CRP (mg/dL)	sIL-2R (U/L)	Histological Diagnosis (Biopsy Site)	Time to Diagnosis	Prognosis	Authors
1	66/F	2 times	Rotatory vertigo, hearing loss	Cardiogenic cerebral embolism	Anticoagulants	Left hemiparesis	782	1.14	1396	Skin, Brain	1 year	N.D.	Mitsutake, et al. [25]
2	63/M	2 times	Floating sensation	Multiple infarction with unknown etiology	Antiplatelets	Left hemiparesis	276	0.08	1910	Skin	2 months	Survival	Hirose, et al. [26]
3	66/F	2 times	Right hearing loss	Paradoxical cerebral embolism	Warfarin	Fever, skin rash	528	0.6	2045	Brain	2 years	Survival	Yamaoka, et al. [27]
4	71/M	2 times	Abnormal behavior	Malignant lymphoma	NA	Seizures	714	8.34	4150	Bone marrow, Lung	3 months	Survival	Tsuda, et al. [28]
5	58/M	3 times	Aphasia	Acute disseminated encephalomyelitis	NA	Diarrhea, adrenal enlargement	597	N.D.	4816	Brain	6 months	Death	Aoyama, et al. [29]
6	79/M	3 times	Cognitive decline	Lacunar infarction	Aspirin and ticlopidine	Right hemiparesis	301	N.D.	N.D.	Brain (via autopsy)	4 months	Death	Usuda, et al. [30]
7	42/M	2 times?	Ataxia, motor aphasia, frontal lobe releasing signs	N.D.	Aspirin	Left hemiparesis	1203	6.4	N.D.	Skin	2 months	Death	Jitpratoom, et al. [31]
8	75/F	2 times	Slurred speech, tonic seizure, facial droop	Hemorrhagic cerebral infarction	NA	Delirium	N.D.	N.D.	N.D.	Brain (via autopsy)	1 month	Death	Haninger, et al. [32]
9	64/M	2 times	Numbness, left leg paresis	N.D.	NA	Left-arm paresis, confusion, prosopagnosia, dysnomia, and color blindness	290	normal	N.D.	Brain	3 months	Death	Lyden, et al. [33]
10	67/M	2 times	Progressive speech difficulties, truncal ataxia	Cerebral emboli by endocarditis	NA	Neurological deficit (unknown detail)	728	3.4	N.D.	Adrenal biopsy	N.D.	Survival	Boslooper, et al. [15]
11	70/F	3 times	Aphasia, right hemianopsia, acalculia, cognitive decline	Ischemic stroke with unknown etiology	Aspirin	Dressing apraxia, body agnosia	N.D.	N.D.	N.D.	Brain	3 months	Death	Hung, et al. [34]
12	65/F	3 times	Disturbed consciousness, left hemiparesis	N.D.	NA	Right hemiparesis, dysarthria, sensory aphasia, right hemianopia	N.D.	N.D.	N.D.	Brain	5 weeks?	Stuporous	Hung, et al. [34]
13	68/M	2 times	Cognitive decline	Aortogenic embolic stroke	Aspirin	Left-half spatial neglect, left hemiplegia	2064	5.7	1462	Brain (via autopsy)	3 months	Death	Ohya, et al. [35]
14	43/F	2 times	Generalized convulsion	Vasculitis	NA	Aphasia	N.D.	N.D.	N.D.	Brain (via autopsy)	9 months	Death	Kenéz, et al. [36]
15	63/F	4–5 times	Numbness in the right upper limb	Lacunar infarction	Aspirin	Confusion, paraplesia	527	N.D.	N.D.	Skin	4–5 months?	Survival	Yunce, et al. [37]
16	47/F	2 times	Rapidly progressive dementia	Inflammatory demyelinating diseases	NA	Left-limb weakness and numbness	338	N.D.	N.D.	Brain	6 months	Death	Wu, et al. [38]
17	75/F	2–3 times	Dysarthria and dysphagia	N.D.	NA	Sensory aphagia, disturbed consciousness	364	N.D.	1540	Brain	4 months	Death	Imamura, et al. [39]
18	67/M	2 times	Seizure	N.D.	NA	Dementia	277	0.8	N.D.	Kidney, Lung (via autopsy)	9 months	Death	Sakamoto et al. [40]
19	56/M	2 times	Cochlear symptoms, right facial paresthesia	N.D.	Aspirin	N.D.	603	N.D.	N.D.	Kidney	1 month	Survival	Popiolek, et al. [41]
20	58/M	2 times	Fatigue, night sweat	N.D.	NA	Gait disturbance, recurrent cramps, dysesthesia in both legs	5843	4.8	N.D.	Bone marrow, muscle	8 months	Death	Bergmann, et al. [42]
Our case	79/M	5 times	Gait disturbance	Lacunar infarction	Clopidogrel	Cognitive decline, double vision, right hemiparesis, and dysesthesia	398	0.75	975	Skin	1 year	Survival	This case

N.D.; no data, NA; not applicable.

## Data Availability

The data supporting the findings of this study are not publicly available due to patient privacy and ethical considerations. Data may be available from the corresponding author upon reasonable request and with appropriate ethical approvals.
